# Cardiac disease-induced trauma and stress-related disorders

**DOI:** 10.1007/s00059-024-05255-0

**Published:** 2024-07-11

**Authors:** Mary Princip, Katharina Ledermann, Rahel Altwegg, Roland von Känel

**Affiliations:** 1 Department of Consultation-Liaison Psychiatry and Psychosomatic Medicine, University Hospital Zurich, University of Zurich, Haldenbachstrasse 16/18, 8091 Zurich, Switzerland; 2 Department of Clinical and Health Psychology, University of Fribourg, Fribourg, Switzerland

**Keywords:** Acute stress disorder, Adjustment disorder, Posttraumatic stress disorder, Cardiovascular disease, Cardiac-induced posttraumatic stress disorder, Akute Belastungsstörung, Anpassungsstörung, Posttraumatische Belastungsstörung, Herz-Kreislauf-Erkrankung, Kardial induzierte posttraumatische Belastungsstörung

## Abstract

**Objective:**

This review aims to present an updated overview of cardiac disease-induced trauma and stress-related disorders such as acute stress disorder (ASD), adjustment disorder (AjD), and posttraumatic stress disorder (PTSD). First, the prevalence of these disorders, their diagnostic criteria, and their differences from other trauma-related disorders are described. Special challenges in diagnosis and treatment are identified, with various screening tools being evaluated for symptom assessment. Additionally, the risk factors studied so far for the development of symptoms of cardiac-induced posttraumatic stress disorder and the bidirectional relationship between posttraumatic stress disorder and cardiovascular diseases are summarized. Various therapeutic interventions, including pharmacological approaches, are also discussed. Finally, various areas for future research are outlined.

**Background:**

Experiencing a cardiovascular disease, particularly a life-threatening cardiac event, can potentially lead to stress-related disorders such as ASD, AjD, and cardiac disease-induced PTSD (CDI-PTSD). If left untreated, these disorders are associated with a worsening cardiac prognosis and higher mortality rates. Approaching treatment through a trauma-focused lens may be beneficial for managing CDI-PTSD and stress-related disorders.

**Conclusion:**

Future research should explore treatment options for both the patients and the caregivers as well as investigate the long-term effects of trauma-focused interventions on physical and mental health outcomes.

## Abstract (de)

**Ziel der Arbeit:**

Ziel der vorliegenden Übersichtsarbeit ist es, einen aktuellen Überblick über traumatische Ereignisse und stressbedingte Störungen im Zusammenhang mit Herzerkrankungen zu geben, mit Fokus auf akuter Belastungsstörung, Anpassungsstörung und posttraumatischer Belastungsstörung. Zuerst wird die Häufigkeit dieser Störungen, ihre diagnostischen Kriterien sowie ihre Unterschiede zu anderen traumaassoziierten Störungen beschrieben. Besondere Herausforderungen in der Diagnose und Behandlung werden identifiziert, wobei verschiedene Screening-Tools zur Bewertung der Symptome evaluiert werden. Darüber hinaus werden die bisher untersuchten Risikofaktoren für die Entwicklung von Symptomen der kardial induzierten posttraumatischen Belastungsstörung und die bidirektionale Beziehung zwischen posttraumatischer Belastungsstörung und kardiovaskulären Erkrankungen zusammengefasst. Auch verschiedene therapeutische Interventionen, einschließlich pharmakologischer Ansätze, werden diskutiert. Abschließend werden verschiedene Bereiche für zukünftige Forschung skizziert.

**Hintergrund:**

Das Erleben einer Herz-Kreislauf-Erkrankung, insbesondere eines lebensbedrohlichen kardialen Ereignisses, kann zu stressbedingten Störungen wie akuter Belastungsstörung, Anpassungsstörung und kardial induzierter posttraumatischer Belastungsstörung führen. Unbehandelt können diese Störungen den Verlauf und die Prognose der kardialen Erkrankung verschlechtern und zu höheren Sterberaten beitragen. Ein auf das Trauma fokussierter Behandlungsansatz könnte sich als nützlich erweisen, um stressbedingte Störungen zu bewältigen.

**Schlussfolgerung:**

Zukünftige Studien sollten effektive und effiziente Behandlungsmöglichkeiten für Patienten und Behandler untersuchen und die langfristigen Folgen von Interventionen mit Fokus auf Traumata für die körperliche und psychische Gesundheit genauer evaluieren.

## Background

Cardiovascular diseases (CVD) and acute cardiac events such as acute coronary syndrome (ACS) continue to be the leading cause of morbidity and mortality worldwide [1]. Although hospitalization rates and mortality from CVD have declined in recent years due to improvements in medical care and the management of risk factors, demographic shifts have resulted in a rise in the incidence of CVD, presenting substantial medical and economic challenges for the healthcare system [2].

A sudden acute cardiac event carries an imminent risk of death and can evoke feelings of anxiety, loss of control, and helplessness, which can potentially be traumatic [3]. Patients suffering from these symptoms may qualify for a formal diagnosis of acute stress disorder (ASD) occurring within 1 month of the cardiac event. Generally, an episode of ASD fades quickly, most often without need for treatment. However, in a substantial number of cases, adjustment disorder (AjD) and/or cardiac disease induced (CDI-) posttraumatic stress disorder (CDI-PTSD) may occur.

Cardiac disease induced-induced PTSD is defined by the presence of multiple clusters of psychological, behavioral, and physiological symptoms, including intrusive thoughts (intrusions), avoidance, negative changes in cognition and mood, as well as increased arousal and stress reactivity [4]. Trauma-related disorders caused by cardiac diseases not only significantly impair quality of life [5] but are also associated with an increased risk of another cardiac event and increased mortality in the first 3 years following the initial event [6].

## Prevalence rates

A significant proportion of CVD patients have stress-related disorders. Table 1 gives an overview of prevalence rates for the three disorders (ASD, AjD, and PTSD). Notably, prevalence rates vary widely across different CVD events [7].

**Table 1:** Prevalence rates of acute stress disorder (ASD), adjustment disorder (AjD), and cardiac-induced posttraumatic stress disorder (CDI-PTSD)

Disorder	Prevalence in CVD patients	Associated factors
ASD	4–34% [3]	Associated with ED readmission [8] Risk factor for long-term survival [9] and CDI-PTSD [10, 11] independent of objective threat, e.g., ruling out ACS [8]
AjD	6–33% [12]	For children, it extends to different forms (e.g., separation anxiety) [13]. Pivotal stress periods identified (e.g., after discharge, after implantation of device) [14]
CDI-PTSD	4–44% [15] 12% average across CVD [7]	Prevalent in 45–48% of partners of CA patients [10] Prevalent in 12–31% of children with cardiac surgery [16]

*ACS* acute coronary syndrome, *CA* cardiac arrest, *CVD* cardiovascular disease, *ED* emergency department

## Diagnostic criteria

Table 2 provides an overview of the symptoms for ASD, AjD, and PTSD based on the tenth revision of the* International Statistical Classification of Diseases and Related Health Problems* (ICD-10; [4]).

**Table 2:** Symptoms of acute stress disorder (ASD), adjustment disorder (AjD), and posttraumatic stress disorder based on ICD-10

Criteria	ASD	AjD	PTSD
*Stressor requirement*	Directly experiencing traumatic event OR witnessing event happen to others OR event happened to close ones Extreme exposure to event details	Identifiable psychosocial stress within the last month	Directly experiencing traumatic event OR witnessing event happening to others OR event happening to close ones Extreme exposure to event details
*Functional impairment*	Symptoms cause clinically significant distress or impairment in social, occupational, or other areas of functioning	Symptoms cause clinically significant distress or impairment in social, occupational, or other areas of functioning	Symptoms cause clinically significant distress or impairment in social, occupational, or other areas of functioning
*Duration of disturbance*	3 days to 1 month post-trauma	No longer than 6 months after the end of the stressful situation	> 1-month duration
*Diagnosis*	Diagnosis must occur within the first month following the traumatic event	Diagnosis can be assigned when symptoms occur within 3 months of the stressor	Diagnosis can be assigned after 1 month has passed since the traumatic event
*Exclusion of other causes*	Symptoms are not attributable to substance use, medication, or another medical condition	Symptoms not attributable to other mental disorders or exacerbation of pre-existing disorder	Symptoms are not attributable to substance use, medication, or another medical condition
*Intrusive symptoms*	Presence of nine or more of the following symptoms from any of the five categories of intrusion, negative mood, dissociation, avoidance, and arousal	Intensive thoughts or memories related to the stressor Lasts ≤ 6 months after the stressor resolves	Presence of more than one intrusive symptom post-event
*Avoidance*	Avoidance of reminders of the traumatic event	Avoidance of reminders of the stressor	Avoidance of trauma-related stimuli
*Alterations in mood and cognition*	Memory issues, negative beliefs, distorted cognitions, negative emotions, decreased interest, detachment, inability to feel positive emotions	Depressed mood, anxiety, difficulty concentrating, changes in appetite or sleep, feelings of hopelessness	Memory issues, negative beliefs, distorted cognitions, negative emotions, decreased interest, detachment, inability to feel positive emotions
*Alterations in arousal and reactivity*	Irritability, recklessness, hypervigilance, startle response, concentration issues, sleep disturbances	Irritability, concentration issues, sleep disturbances, alterations in appetite, recklessness, physical symptoms (such as headaches or stomachaches)	Irritability, recklessness, hypervigilance, startle response, concentration issues, sleep disturbances

*ICD-10* tenth revision of *the International Statistical Classification of Diseases and Related Health Problems*

## Differences from other trauma-related disorders

Post-traumatic stress disorder (PTSD) induced by a medical event can exhibit several pertinent differences from other trauma-related disorders [17]:

1. *Source of threat:* Cardiac trauma-related disorders stem from internal threats originating from within one’s own body, such as heart-related events, unlike traditional trauma that typically involves external causes like accidents, assaults, or disasters.
2. *Nature of intrusive symptoms:* Intrusive symptoms in cardiac trauma-related disorders can manifest as present or future-oriented intrusions, often referred to as “flashforward intrusions.” These typically revolve around fears of experiencing another cardiac event or concerns about mortality due to heart-related issues. By contrast, traditional trauma-related disorders may involve intrusions related to the traumatic event itself, typically in the form of flashback intrusions.
3. *Avoidance behaviors:* Patients with cardiac trauma-related disorders may exhibit avoidance behaviors toward health-promoting activities like physical exercise, medical appointments, or medication adherence due to fear of exacerbating their condition. This avoidance may result in heightened anxiety and guilt. In traditional trauma-related disorders, avoidance behaviors may be more focused on avoiding reminders or triggers of the traumatic event.
4. *Interpretation of physical symptoms:* Individuals with cardiac trauma-related disorders may misinterpret physical sensations such as increased heart rate or shortness of breath as signs of impending cardiac issues, leading to heightened anxiety and hyperarousal. This contrasts with traditional trauma-related disorders where physical symptoms may be more directly associated with triggers or reminders of the traumatic event.

## Challenges in diagnosis and treatment

Distinguishing between physical symptoms of cardiac trauma-related disorders and symptoms of actual heart disease can be challenging for both the patients and the clinicians, potentially leading to prolonged anxiety and difficulty in initiating appropriate treatment. Tailored interventions that address the unique challenges of cardiac trauma-related disorders may be necessary for effective management. These differences highlight the distinct nature of trauma-related disorders stemming from cardiac events compared to those arising from traditional traumatic experiences, necessitating specialized approaches in diagnosis and treatment [18].

## Screening tools

### ICD-11 and the “Adjustment Disorder–New Module”

The diagnosis of AjD typically depends on the clinical judgment of a professional, as there is no established gold standard for standardized assessment. The new ICD-11 diagnostic criteria for AjD support a uni-faceted approach with core symptoms and additional features, resulting in a clearer diagnostic concept [19]. Central symptoms include preoccupation with the stressor and an inability to adapt. Signs of preoccupation encompass recurrence, distressing thoughts, or persistent rumination about the stressful event. Failure-to-adapt symptoms may manifest as sleep disturbances or difficulties in concentration. In accordance with this revised understanding of AjD, a self-reported assessment tool called the “Adjustment Disorder–New Module” (ADNM) was validated. So far, only a few studies have used the new ICD-11 criteria for AjD in cardiac patients, concluding that the new questionnaire is effective and recommending it for use in future studies [20, 21].

### Primary Care PTSD Checklist

Screening for and diagnosing PTSD in cardiac patients can be challenging due to the unique symptomatology, such as intrusive thoughts about future cardiac events. Presently, there is no dedicated diagnostic tool designed for evaluating CDI-induced PTSD symptoms. International and scientific clinical guidelines advocate for the screening of all cardiac patients for psychological distress following such an event [22]. Early recognition and intervention can significantly improve outcomes for these patients [22]. The Primary Care PTSD Checklist (PC-PTSD) can be used to screen for PTSD in cardiac patients [23]. The PC-PTSD‑5 is effectual in identifying potential PTSD symptoms on an individual basis. The questions about the individual symptoms of PTSD should refer to the cardiac event as the traumatic experience. Patients who test positive on the screening require further assessment, ideally conducted through structured interviews.

## Risk factors for the development of CDI-PTSD

Numerous studies indicate that risk factors for CDI-PTSD following a cardiac event encompass various domains. These include sociodemographic factors (such as female gender, younger age, and potentially ethnic minority status and low education), psychological characteristics (including prior psychological issues, high intrusion symptoms, and severity of ASD and depression symptoms), and aspects related to the cardiac event itself (such as perception of life threat, history of referral to a psychologist, and the context of care during the event; [24, 25]). Of particular importance is an individual’s perception of illness following a cardiac event, which consistently influences the development of CDI-PTSD symptoms [26]. Specifically, intense pain, fear, and helplessness in response to the ACS significantly heighten the likelihood of PTSD development [27]. Contradictory results have been reported with the use of benzodiazepines, as sedation in the initial phase of resuscitation reduced the risk of developing PTSD [28]. However, patients with an ACS and benzodiazepines had an increased risk of PTSD [29]. Additional risk factors for the development of these symptoms include environmental and treatment-related factors (e.g., chaotic hospital admissions, treatment complications, staff statements; [29]), personality traits (Type D, neuroticism, hostility, alexithymia; [11]), and a patient’s biopsychosocial history (stressful life events, prior heart disease, other somatic conditions; [30]). Cardiac patients treated during emergency department overcrowding, hallway care, and perceived inadequate clinician–patient communication also appear to be at greater risk for subsequent PTSD [31]. Interestingly, objective indicators of cardiac injury, such as troponin T levels in ACS, show little correlation with subsequent PTSD symptoms [27]. Conversely, social support and resilience factors (such as internal control beliefs, humor, patience, and repressive coping strategies immediately after the traumatic event) have been shown to have a protective effect against the development of CDI-PTSD symptoms [24].

## The bidirectional association between PTSD and CVD

The bidirectional association between PTSD and CDI-PTSD has long been linked to an increased risk of CVD, although the precise reciprocal relationship between PTSD and CVD remains not fully understood [32]. Several physiological mechanisms have been investigated to elucidate the link between PTSD and CVD, including autonomic dysfunction, disruptions in neuroendocrine regulation, and inflammatory processes (for details, refer to [33]). Various neurobiological underpinnings may be involved, consistent with the conceptualization of PTSD as a disorder characterized by altered formation and/or extinction of emotional memories, along with dysregulated responses to threat and stress [34]. These encompass alterations in the functioning and connectivity of specific brain regions crucial for emotional processing and cognition, notably the amygdala, insula, dorsal anterior cingulate, and ventromedial prefrontal cortex [34].

## Therapeutic interventions

Preventing cardiac-induced stress-related disorders involves addressing both the physical and psychological aspects of cardiac events. Since not all cardiac events can be prevented, it is important to note that several strategies can help reduce the risk and mitigate the impact of traumatic experiences related to these events. Given that the personal experience of stress and discomfort is more important than the severity of the cardiac condition [7], establishing a sense of safety and control is essential.

An immediate one-time trauma-specific counseling shows no beneficial long-term effect on preventing posttraumatic stress compared with one session of general stress counseling [35, 36]. However, trauma-specific counseling was more effective in patients who perceived an increased level of social support [37]. After a cardiac event, there are several starting points for preventive interventions based on the aforementioned risk factors. Providing patients and their families with information and education about cardiac conditions, treatment options, and coping strategies could help reduce anxiety, fear, and distress. A multidisciplinary approach has been emphasized to support ICD patients with shock experience and provide detailed and practice-friendly psychoeducation [38]. Furthermore, web-based cognitive behavioral therapy (CBT) has been recommended as a low-threshold alternative to in-person treatments for centers lacking comprehensive cardiac psychology services.

This goes in line with therapeutic options for AjD. Interventions usually aim at three factors: (a) elimination or mitigation of stress (e.g., treatment of underlying CVD); (b) improvement of coping and adaptation (e.g., taking up activities); and (c) symptom reduction and behavioral change (e.g., CBT for anxiety/depression; [39]). As AjD is by definition transient, the authors propose a stepped approach with level 1 watchful waiting, which could be accomplished during cardiac rehabilitation. Level 2 consists of low-intensity psychological interventions such as web-based programs. For those patients who do not benefit sufficiently, level 3 recommends individual psycho- and/or pharmacotherapy. Level 4 includes inpatient treatment for people with severe AjD with, for instance, suicidal tendencies [39]. A recent review and meta-analysis reported web-based interventions for AjD areas to be effective [40]. One example is the Internet-based modular program Brief Adjustment Disorder Intervention (BADI) for ICD-11-AjD [41]. The BADI is a CBT-based program and consists of four modules: relaxation, time management, mindfulness, and strengthening relationships. It showed promising effects on AjD when used at least once in 30 days [42]. Therapeutic support did not enhance its effectiveness [43]. Participation in cardiac rehabilitation following a cardiac event can help individuals recover physically and emotionally. These programs typically encompass supervised exercise training, educational sessions promoting heart-healthy lifestyle modifications, and counseling or support groups to address psychological concerns. However, individuals with PTSD or AjD exhibit reduced participation in physical activity and tend to avoid their physical limits [21, 44]. As the occurrence of somatic symptoms such as chest tightness or heartbeat symptoms can increase post-traumatic stress [45], this could culminate in a self-perpetuating cycle of avoidance with mutually reinforcing factors. Exercise, however, has a beneficial effect on PTSD, whether used alone or as a complement to conventional therapy [46].

Despite the undeniable indication for therapeutic intervention, a study reported only 30–40% of patients with diagnosed CDI-PTSD received some form of treatment, whether medication or counseling [47]. Trauma-focused psychotherapy is more beneficial than non-trauma-focused therapies in reducing PTSD severity [48]. Meta-analyses have demonstrated encouraging findings regarding the efficacy and long-term impact of exposure-based therapies such as prolonged exposure (PE), trauma-focused CBT, cognitive therapy (CT), and eye movement desensitization and reprocessing (EMDR; [49]) as well as narrative exposure therapy (NET; [50]). Imaginal exposure therapy (IET) proved safe, as evidenced by the absence of significant changes in blood pressure and heart rate during exposures [51]. Promising approaches extend beyond traditional psychotherapeutic interventions. Innovative therapies such as biofeedback may serve as a variation of exposure therapy [52].

## Pharmacotherapy

In addition to psychotherapy, psychotropic drugs are another option for treatment of severe PTSD. Selective serotonin reuptake inhibitors (SSRIs) and serotonin-norepinephrine reuptake inhibitors (SNRIs) are the drugs of choice [53]. Of the SSRI group, sertraline and citalopram seem safe for patients with CVD [54]. Tricyclics should be avoided in patients with CVD [15]. Furthermore, clinicians should monitor possible cardiometabolic side effects (e.g., weight gain) and discuss the risks and benefits of the medication with patients before and throughout the treatment [53]. Benzodiazepines exhibit a short-term anxiolytic effect, yet they increase the risk of developing PTSD symptoms after an ACS, making it advisable to use them with caution [29]. An important contraindication arises concerning patients with heart failure, as antidepressants have been associated with an increased risk of all-cause mortality [55]. Antidepressants, i.e., SSRIs, should therefore be avoided in favor of psychotherapy [56]. There is only limited research on pharmacotherapy for AjD, but studies suggest caution against the use of benzodiazepines [57].

## Future research

Future research should aim to better identify early signs of CDI-induced distress with screening for ASD, especially in patients with a history of mental illness, as they seem most vulnerable to additional stress [58]. Clinician–patient communication is a critical factor that can influence the development of PTSD symptoms [59]. As the perception of a hectic hospital environment and crowded emergency department are associated with an increased risk for ASD and PTSD and can lead to a higher re-admission rate [60, 61], interventions should go beyond individual care and address structural factors, too. Future research on effective interventions for ASD should aim to help patients with cardiac disease mitigate their distress and perceived threat while concurrently preventing the emergence of long-term cardiac trauma and stress-related disorders such as AjD and PTSD. Not all patients benefit from the same intervention [37].

Further research is needed to identify specific risk factors, enabling more personalized interventions that effectively target the most vulnerable patients and those who benefit the most. A promising differentiation has been offered in a recent study of ACS patients that identified three clusters associated with posttraumatic stress and depression [62]: (a) low-risk cluster with the highest resilience, task-oriented coping, positive affect, and social support; (b) a lonely cluster with the lowest social support and resilience; and (c) avoidant cluster with the lowest task-oriented coping and positive affect. Future research is needed to assess whether addressing these factors may yield protective benefits. Research on early intervention after a traumatically experienced medical event to prevent PTSD is promising, albeit preliminary and limited [63], but is even more sparse regarding AjD. One barrier is the unclear defined criteria and the lack of assessment methods for AjD [64]. The ICD-11 introduced a major change in the definition of AjD with two core symptoms: preoccupation and failure to adapt [65]. Increasing evidence supports the concept [66] and the assessment tool (ADNM; [19]). With the establishment of standardized criteria and assessment tools, future studies can more reliably assess the efficacy of preventive and early intervention programs for AjD.

## Conclusion

The development of targeted interventions for the unique challenges posed by trauma-related disorders in the context of cardiac events, including adjustment disorder and cardiac disease-induced posttraumatic stress disorder, is crucial. These interventions should aim to address the specific symptomatology, risk factors, and barriers to treatment engagement associated with cardiac trauma-related disorders, thus improving patient outcomes and quality of life. The integration of psychological support into standard cardiac care pathways, such as cardiac rehabilitation programs, may help ensure that patients receive timely and appropriate interventions to address their psychological needs alongside their physical recovery.

## Declarations

For this article no studies with human participants or animals were performed by any of the authors. All studies mentioned were in accordance with the ethical standards indicated in each case.
